# Nanotubes Make Big Science

**DOI:** 10.1371/journal.pbio.0020215

**Published:** 2004-07-13

**Authors:** Fabio Demontis

## Abstract

Tiny protrusions on the surface of cells might be a more common mechanism for cell communication than previously expected

During development, cells need to communicate with each other to establish properly organised and functional tissues. Cells communicate with each other in various ways, such as by secreting and receiving diffusible molecules (morphogens, hormones, and neurotransmitters) or by establishing intercellular connections (gap junctions and cell protrusions) to allow a direct exchange of instructive factors. A recent paper has shown that communication via tiny cell protrusions might be a more common mechanism than previously expected (Rustom et al. 2004).

Many different types of cell extensions have been described in a variety of developmental processes and organisms (Miller et al. 1995; Bryant 1999; Chou and Chien 2002; Rorth 2003), and for most of them a role in cell-to-cell communication has been hypothesized. For example, in the mouse, Salas-Vidal and Lomeli (2004) have described long processes (filopodia) that connect tissues in early embryos. Because these protrusions contain receptors for some well-known signalling molecules, it is thought that they might be responsible for receiving signals from neighbouring cells. Similarly, it has been proposed in the development of the Drosophila wing and eye imaginal discs (precursors of adult structures) that signals modulating the growth and patterning of one epithelial layer of cells are received through microtubule-based cell extensions arising from the apposing epithelium (Cho et al. 2000; Gibson and Schubiger 2000). Furthermore, in the wing imaginal disc, planar extensions called cytonemes arise from the periphery of the epithelium and grow towards a central area in the wing disc that produces the signalling molecule Decapentaplegic. This directionality of growth, and the observation of vesicles inside cytonemes, led Ramirez-Weber and Kornberg (1999) to propose that cells meant to receive a signal were searching actively for it, extending long cell protrusions towards the region from which signals were emanating.

Although cell protrusions have been described in different developmental processes, tissues, and organisms, their potential role in cell signalling has been difficult to pin down. Most cell processes are very fragile, and their study is mainly limited to live tissues; in these conditions it is technically challenging to define how the signalling is mediated via protrusions. Possibly, it could occur through the release of free molecules, in a similar manner to synaptic neurotransmission, or shedding of vesicles as exosomes followed by endocytosis by the recipient cell. Alternatively, membranetethered ligands (such as Delta) on the protrusion could bind and activate receptors displayed on the surface of the receiving cell (De Joussineau et al. 2003).

The paper by Rustom and colleagues has provided a new outlook on the role of cell protrusions, by reporting a novel mechanism employed to transmit signals between cells connected by a protrusion. Surprisingly, they did not observe any of the mechanisms described above. Rather, transfer of molecules and organelles occurred directly from the cytoplasm of one cell to the other, passing through a protrusion that established membrane continuity between the connected cells.

Using rat PC12 cells, Rustom and colleagues observed ultrafine protrusions (with a diameter of only 50–200 nm and a length spanning several cell diameters) connecting sparse cells in culture (Figure 1). Similar to other cell protrusions, these structures, termed tunnelling nanotubes (TNTs), displayed a pronounced sensitivity to both mechanical stress and chemical fixation and even to prolonged light excitation, resulting in the rupture of many of them. TNTs are actin-based and devoid of microtubules: interestingly most other types of cell protrusions also contain actin (Condeelis 1993; Rorth 2003). The researchers also confirmed the existence of TNTs in a human cell line (human embryonic kidney cells) and rat primary cells (normal rat kidney cells), suggesting that TNTs are not a peculiarity of PC12 cells.

**Figure 1:**
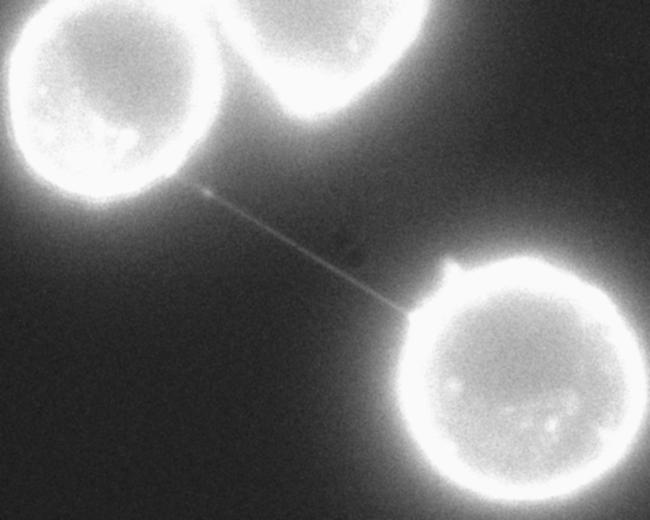
A TNT Connecting Two Neighbouring Cells Immunofluorescence analysis of wheat germ agglutinin–stained PC12 cells that shows a TNT, a novel type of cell protrusion that establishes membrane continuity between two neighbouring cells. Transfer of molecules and organelles can occur directly from the cytoplasm of one cell to that of the other. (Image courtesy of Hans-Hermann Gerdes.)

Aiming to investigate how TNTs were linking cells, the authors performed scanning and transmission electron microscopy of TNTs. They observed a seamless transition between TNTs and the cells they were connected to, suggesting that indeed there was continuity between the membranes of the two connected cells. Rustom and colleagues then went on to test whether TNTs could be used to transmit signals between cells. The experimental approach used was to mark two populations of cells in a distinct way, either by introducing genes that encoded proteins tagged with green fluorescent proteins or by using dyes. The two different cell populations were then mixed, cocultured, and analysed for transfer of marked proteins or dye-stained organelles from one cell to another, between cells that were differently marked and connected by a TNT. Strikingly, soluble cytoplasmic molecules could not pass freely along the TNTs (with actin tagged with green fluorescent protein being the only exception), whereas membrane-bound proteins were transferred along TNTs and detected in the receiving cells, further supporting the likelihood of membrane continuity between connected cells. Rustom and colleagues also observed transport of vesicles, which seemed to be unidirectional. Finally, in transfer experiments performed at close to 0 °C, where endo-, exo-, and phagocytosis would be blocked, vesicle exchange still occurred, suggesting that these events are not required for vesicle transfer and further supporting the idea that membrane continuity exists between connected cells. By contrast, interfering with actin polymerization, using the drug latrunculin-B, led to protrusion removal and arrest in organelle transfer, indicating that actin is required both for protrusion biogenesis and organelle transport.

Taken together, the experiments performed by Rustom and colleagues strongly suggest a role for cell protrusions in cell-to-cell communication. They also provide evidence, in culture, for a novel mechanism used by cell protrusions to transport molecules and organelles. It will be interesting to test whether TNTs also exist in living tissues and, if so, what molecules they transport. TNTs could be distinct from the protrusions known so far and could be responsible for establishing another type of connection between cells. They could connect all cells in a tissue, directly or indirectly, establishing a global interaction network potentially important in exchanging basic survival information as well as positional cues (Milan et al. 2001).

Another interesting question is how connections such as TNTs are established. Rustom et al. have shown that, initially, many filopodial extensions arise from one cell and are directed toward a neighbour. As soon as one of them reaches the target, it is stabilised, while the others degenerate. It is possible that membrane fusion occurs between the tip of the protrusion and the planar plasma membrane of the target cell. However, membrane fusion can be more easily achieved if the tips of two cell protrusions fuse with each other, thus suggesting the participation, in the process of membrane fusion, of microvilli or other tiny protrusions belonging to the target cell. Fusion between two protrusions is reported to rely on the cylindrical shape and narrow diameter of cell protrusions and also on the localised concentration of adhesion/fusion molecules at the tips of the cell protrusions, such as microvilli, that display particular tip-specific membrane microdomains (Monck and Fernandez 1996; Wilson and Snell 1998; Roper et al. 2000). The work performed by Rustom and colleagues suggests that cell protrusions are a general mechanism for cell-to-cell communication and that information exchange is occurring through the direct membrane continuity of connected cells, independently of exo- and endocytosis. It is important to determine whether events similar to these seen in cell culture are occurring in tissues and what functions cell protrusions perform during tissue morphogenesis.

In my work as a graduate student, I am trying to address this question. We need to identify the types of cell protrusions that are present in tissues and the molecular complexes localizing on them as well as their functions. To then prove that cell protrusions are important in cell-to-cell communication in tissues, we would need to remove the protrusions and see how this affects tissue architecture and function. However, the necessary tools are still missing, given the lack of knowledge of the specific molecules important for the biogenesis of these protrusions. Thus far, the function of cell protrusions has been hypothesized mainly on the basis of their location in tissues and on crude attempts to remove them, for example by altering the actin cytoskeleton or even by removing the entire epithelium they belong to.

The paper by Rustom et al. has shed some new light on these still mysterious cellular arms and has further boosted my interest in this emerging field of cell and developmental biology.

## Abbreviation

TNT: tunnelling nanotube
